# Recent Advances and Future Perspective of DC-Based Therapy in NSCLC

**DOI:** 10.3389/fimmu.2021.704776

**Published:** 2021-06-28

**Authors:** Iris A. E. van der Hoorn, Georgina Flórez-Grau, Michel M. van den Heuvel, I. Jolanda M. de Vries, Berber Piet

**Affiliations:** ^1^ Department of Pulmonary Diseases, Radboud University Medical Center, Nijmegen, Netherlands; ^2^ Department of Tumor Immunology, Radboud Institute for Molecular Life Sciences, Radboud University Medical Center, Nijmegen, Netherlands

**Keywords:** dendritic cells, lung cancer, immunotherapy, immunology and lung cancer, non-small cell lung cancer

## Abstract

Current treatment for patients with non-small-cell lung cancer (NSCLC) is suboptimal since therapy is only effective in a minority of patients and does not always induce a long-lasting response. This highlights the importance of exploring new treatment options. The clinical success of immunotherapy relies on the ability of the immune system to mount an adequate anti-tumor response. The activation of cytotoxic T cells, the effector immune cells responsible for tumor cell killing, is of paramount importance for the immunotherapy success. These cytotoxic T cells are primarily instructed by dendritic cells (DCs). DCs are the most potent antigen-presenting cells (APCs) and are capable of orchestrating a strong anti-cancer immune response. DC function is often suppressed in NSCLC. Therefore, resurrection of DC function is an interesting approach to enhance anti-cancer immune response. Recent data from DC-based treatment studies has given rise to the impression that DC-based treatment cannot induce clinical benefit in NSCLC by itself. However, these are all early-phase studies that were mainly designed to study safety and were not powered to study clinical benefit. The fact that these studies do show that DC-based therapies were well-tolerated and could induce the desired immune responses, indicates that DC-based therapy is still a promising option. Especially combination with other treatment modalities might enhance immunological response and clinical outcome. In this review, we will identify the possibilities from current DC-based treatment trials that could open up new venues to improve future treatment.

## Introduction

Lung cancer is the leading cause of cancer-related death worldwide (1). This type of cancer is a heterogeneous disease (2). Based on histology, lung cancer is divided into small-cell lung cancer (SCLC) and non-small-cell lung cancer (NSCLC). NSCLC is the most prevalent form, accounting for about 80-85% of the lung cancer cases (3). The five-year overall survival rate (OS) for NSCLC is around 20% in the western world, highlighting the importance to explore the current and future therapeutic approaches in this field (4, 5).

Although surgery remains the cornerstone of therapy for early-stage NSCLC, a wide range of therapeutic options for adjuvant treatment or treatment of advanced stage disease have been introduced over the last decade. Targeted therapy and immunotherapy are examples of these novel therapies (6, 7). Targeted therapy targets specific alterations in NSCLC cells that stimulate tumor growth, for example mutations in the epidermal growth factor receptor (EGFR). Many other specific targets in NSCLC have been identified over time. Targeted therapy often leads to prolonged survival and greatly enhanced quality of life in this subgroup of patients (8, 9). Most patients with NSCLC lack actionable therapy targets. Therefore, immunotherapy, with or without chemotherapy, is the first-line treatment for the majority of NSCLC patients with advanced stage disease. Although targeted therapy and immunotherapy greatly improved clinical outcome in NSCLC, not all patients respond (10). Moreover, the patients who do respond eventually develop therapy resistance. Therefore, a high clinical need for new systemic treatment modalities remains. During this review we will shine our light on the rather unexposed field of dendritic cell (DC)-based therapies, to explore whether these could be a valuable treatment option for NSCLC.

## Immunotherapy in NSCLC

The role of the immune system in prevention of cancer development and progression has been widely recognized. Immunotherapy exploits this role by stimulating the patient’s immune system to eliminate the tumor. Different immunotherapeutic strategies are being used or currently studied for their use in cancer. These can be largely subdivided into cancer vaccines, cellular therapies, immune stimulatory agonists and immune checkpoint inhibitors (ICIs). NSCLC is a promising potential target for immunotherapeutic approaches due to its high tumor mutational burden, which enhances immunogenicity of the tumor (11). ICIs are the only currently approved immunotherapy option for NSCLC. The most frequently used ICIs are directed against programmed-death receptor 1 (PD-1), expressed on immune effector cells such as T cells and natural killer (NK) cells, or its ligand programmed-death ligand 1 (PD-L1), which is expressed on antigen-presenting cells (APCs) and tumor cells. Receptor binding of PD-1 can lead to inhibition of effector cell function and survival, while it induces T regulatory cells (Tregs) (12–14). Immune cells in tumors frequently demonstrate a non-functional or ‘exhausted’ phenotype which hampers an anti-cancer immune response. ICIs aim to revert this immunosuppressive phenotype, thereby inducing an efficient anti-cancer immune response (15).

Recently, anti-cytotoxic T-lymphocyte associated protein 4 (CTLA-4), which is another ICI, has been registered to be used in combination with a PD-1 inhibitor in the United States and is registered for the combination with a PD-1 inhibitor and chemotherapy in Europe (16, 17). CTLA-4 is expressed on T cells after activation. It is also constitutively expressed on Tregs. CTLA-4 binds CD80 and CD86 on APCs. Receptor binding transmits an inhibitory signal to the T cell. Furthermore, binding of CTLA-4 to CD80 and CD86 blocks their binding to T cell receptors thereby hampering T cell activation (18).

Nowadays, it is known that the tumor and its surrounding microenvironment (TME) can modulate anti-tumor immune responses. Recent data suggest that the low response rate to ICIs could be partly explained by the lack of immune cells in the TME or other regulatory factors that prevent an anti-tumor immune response (19). Therefore, other forms of immunotherapy to enhance the anti-tumor immune response are currently being studied, such as DC-based therapy.

## DC-Based Therapy

DC-based therapy depends on the fundamental link that DCs form between tumor antigen recognition and an anti-tumor immune response. More specifically, DCs are highly specialized APCs that show the highest antigen-presenting potential when inducing naïve T cell activation (20). In tissue, DCs are constantly scanning their surroundings. Upon antigen encounter in the presence of pathogen-associated molecular patterns (PAMPs) or damage-associated molecular patterns (DAMP), DCs get activated, undergo maturation and secrete large amounts of pro-inflammatory cytokines to shape the local inflammatory environment (6, 21, 22). After maturation, DCs migrate to the lymph node where they activate T cells to induce an immune response directed against their presented antigen. In absence of PAMP or DAMP signals during antigen encounter, DCs remain immature, migrate to the lymph nodes and induce antigen-specific tolerance in T cells. DC-based therapies showed promising results in several malignancies such as melanoma, prostate cancer, and glioma (23–25). In NSCLC only early-phase clinical trials have been performed, which show disappointing clinical results but were not powered to evaluate clinical effect. In this review, we will analyse these studies and discuss different possibilities to optimize DC-based therapy in order to improve therapeutic effects.

## DC Vaccination Monotherapy for NSCLC

The DC-based therapy in NSCLC consists of the vaccination of patients with DCs. In all studies investigating DC vaccination in NSCLC patients, monocytes derived from autologous peripheral blood mononuclear cells (PBMCs) were differentiated to moDCs *ex vivo* (**Table 1**) (26–36). These DCs were then primed with a combination of several synthetic peptides of commonly expressed tumor antigens in NSCLC or autologous tumor lysate. In the majority of studies, primed DCs were administered *via* multiple subcutaneous injections. *In vivo*, these DCs are supposed to activate cytotoxic T cells that will induce a tumor-directed immune reaction.

**Table 1:** Characteristics of studies in which DC vaccination monotherapy was performed in NSCLC.

Ref.	Subject number	Clinical stage	Antigen source	DC maturation status	Type and regimen of DC administration	Most important results after vaccination
**Ueda et al., Int. J. Oncol. (** 26 **)**	N = 3	III and IV^a^	CEA peptide	Immature	5 biweekly i.d. and s.c. vaccinations	2 out of 3 patients showed a DTH response.
**Hirschowitz et al., J. Clin. Oncol. (** 27 **)**	N = 16	I, II and III	Irradicated tumor lysate or lysate of a NSCLC cell line	Mature	2 i.d. vaccinations	6 out of 16 patients showed tumor-specific IFN-γ T cell responses. No correlation between immunological response and OS or DFS was determined.
**Chang et al., Cancer (** 28 **)**	N = 6	III and IV	Tumor lysate	Not fully mature^b^	4 weekly vaccinations followed by 2 biweekly boost vaccinations in the inguinal lymph nodes	2 out of 6 patients demonstrated increased tumor-specific IFN-γ T cell responses. These 2 patients demonstrated stable disease.
**Hirschowitz et al., Lung cancer (** 29 **)**	N = 14	I, II and III	Irradicated tumor lysate or lysate of a NSCLC cell line	Immature	2 i.d. vaccinations	10 out of 14 patients showed tumor-specific IFN-γ T cell responses. No correlation between immunological response and OS or DFS was demonstrated.
**Um et al., Lung cancer (** 30 **)**	N = 9	IIIB and IV^a^	Tumor lysate	Mature	3 i.d. DC vaccinations at 2 weeks interval	5 out of 9 patients showed increased tumor-specific IFN-γ T cell responses. All patients demonstrated disease progression.
**Perroud et al., J. Exp. Clin. Cancer Res. (** 31 **)**	N = 5	III and IV	Peptides of WT-1, MAGE-1, and Her-2/neu	Unknown^c^	2 biweekly s.c. and i.v. vaccinations	*Ex vivo*, T cell responses directed towards the DC vaccine were increased. 2 out of 5 patients showed an unexpectedly long OS.
**Engell-Noerregaard et al., World J. Of Vaccine (** 32 **)**	N = 22	III and IV	Lysate of a melanoma cell line expressing among others MAGE-A/B	Mature	A weekly s.c. vaccination for 5 weeks, followed by a booster vaccination after 6 weeks, with s.c. IL-2, COX-2 inhibitors, and TLR7 agonist^d^	Ex vivo, vaccination-specific IFN-γ T cell responses were mostly observed in patients showing stable disease. Some patients showed unexpected long survival.
**Takahashi et al., Eur. J. Cancer (** 33 **)**	N = 47	II, III and IV^e^	Tumor lysate or multiple peptides of WT-1, MUC1, and CEA	Immature	≥ 1 biweekly s.c. vaccination^f^	Patients who received WT-1 vaccine showed increased OS.
**Takahashi et al., Cancer Immunol. Immunother. (** 34 **)**	The above study group was extended to N = 240	II, III and IV^b^	WT-1 and/or MUC-1 peptide	Immature	≥ 5 biweekly s.c. vaccinations^f^	Having a DTH response was correlated with increased survival. No difference in OS between patients vaccinated with WT-1 DCs and patients vaccinated with other DC vaccines was determined.
**Ge et al., BMC Cancer (** 35 **)**	N = 15	I, II and IIIA	Survivin and MUC-1 peptides^g^	Partly mature^h^	3 weekly i.v. vaccinations	Circulating Tregs were significantly decreased 2 weeks after vaccination. Improved quality of live was reported.
**Li et al., Oncol. Lett. (** 36 **)**	N = 16	I, II and III	MAGE-A3 and Survivin peptides	Mature	16 rounds of two monthly i.d. vaccinations	All patients showed a DTH response. In 15 out of 16 patients, tumor-specific IFN-γ T cell responses were increased.

Studies are displayed in order of publication (old to new). ^a^Patients without response to first-line treatment or who declined first-line treatment were included, ^b^DCs were HLA-DR^+^CD86^+^CD40^+^CD80^low^CD83^-^CCR7^-^, ^c^No established maturation method was used and no data that showed the maturation status of the DCs was available, ^d^When patients showed no disease progression after vaccination, 1 boost vaccination per 4 weeks was administered, ^e^Patients who had inoperable tumors or relapsed quick after surgery, ^f^When patients showed no disease progression, vaccination was repeated. ^g^DCs were also incubated with inhibitors of suppressor of cytokine signalling 1 (SOCS1), ^h^DCs were HLA-DR^+^CD80^+^CD83^+^CD86^+^CD40^+^CD14^-^CCR7^-^; MUC-1, Mucin-1; CEA, carcinoembryonic antigen; i.d., intradermal; s.c., subcutaneous; DTH, delayed-type hypersensitivity; WT-1, Wilms’ tumor protein 1; MAGE-1, melanoma-associated antigen 1; her-2/neu, human epidermal growth receptor 2; i.v., intravenous; IFN, interferon; Tregs, T regulatory cells; IL-2, interleukin 2; COX-2, cyclo-oxygenase 2; TLR-7, Toll-like receptor 7; DFS, disease-free survival.

All studies showed DC vaccination to be safe. In addition, many studies examined vaccine-specific immunological responses by determining *ex vivo* T cell responses directed towards the vaccine or by performing a delayed-type hypersensitivity (DTH) test. The principle of a positive DTH test is that if T cells are activated by DC vaccination, this DC vaccine will be recognized upon injection in the skin. This will cause a local immune reaction resulting in erythema. Most studies initially confirmed expression of their used tumor antigen in the tumor or used autologous tumor lysate for DC priming. Interestingly, vaccine-specific immunological responses were demonstrated in most studies after vaccination. However, this induced tumor-specific immune response was almost never linked to a radiological response or improved survival.

## DC Vaccination Combination Therapies for NSCLC

Investigators have also been focusing on the effect of combining DC vaccination with other therapies. For instance, chemotherapy and radiotherapy are hypothesised to enhance anti-tumor immunity and could therefore synergize with immunotherapy. A well-described effect of chemotherapy and radiotherapy is immunogenic cell death of cancer cells, exposing high levels of tumor antigen and DAMP molecules to immune cells in the TME (37–39). The superior immune-activating ability that chemotherapy and radiotherapy induce in cancer cells is highlighted in DC vaccination studies of cancer mouse models exploiting this strategy. In these studies, DC vaccination of DCs loaded with radiation-treated or chemotherapy-treated cancer cells, resulted in reduced tumor volume compared to mice vaccinated with DCs loaded with untreated tumor cells (40–43). Moreover, chemotherapy and radiotherapy were reported to stimulate human leukocyte antigen I (HLA-I) expression of the tumor, making tumor cells more sensitive to cytotoxic killing by CD8^+^ T cells (38). It is important to define the optimal dose of chemotherapy or radiotherapy for combination treatment with immunotherapy, as high doses of chemo- and radiotherapy can induce cell death of immune cells as well (37). The synergistic effect of chemotherapy to DC vaccination was recently validated in a human melanoma study (44). For NSCLC, this synergistic effect of both chemotherapy and radiotherapy with DC vaccination was confirmed in mouse models (45–47). In human NSCLC, only one study examined the combination of chemotherapy with DC monotherapy, but many studies investigated the combined effect of chemo- and/or radiotherapy, DC vaccination and cytokine-induced killer cells (CIK) (**Table 2**) (48, 50–55). CIK cells consist of a heterogeneous group of T cells, NK cells, and NKT cells. CIK cells are derived from autologous PBMCs, activated and expanded *ex vivo* under influence of anti-CD3 and cytokines, such as interferon γ (IFN-γ) and interleukin 2 (IL-2) (56). CIK therapy was shown to be safe and had a response rate of 39% in various tumors. Moreover, CIK treatment was associated with increased survival (57). Co-culture of CIK cells and DCs enhanced cytolytic function of CIK cells and increased both IL-12 secretion by DCs and levels of immunostimulatory receptors on DCs as well as CIK cells (58).

**Table 2:** Characteristics of studies in which DC vaccination combination therapy was performed in NSCLC.

Ref.	Subject number	Clinical stage	Antigen source	DC maturation status	Type and regimen of DC administration	Most important results after vaccination
**Zhong et al., Cancer Immunol. Immunother. (** 48 **)**	N = 28 (DC-CIK + chemotherapy = 14; chemotherapy = 14)	III and IV	CEA peptide	Immature	All patients received 4 cycles of vinorelbine with cisplatin chemotherapy. The DC-CIK + chemotherapy group in addition received 4 monthly cycles of i.v. DC-CIK vaccinations.	Patients in the DC-CIK + chemotherapy group demonstrated significantly increased PFS compared to the chemotherapy only group. There was no difference between 1-, 2-, and 5-year OS between the different groups.
**Shi et al., J. Immunother. (** 49 **)**	N = 54 (erlotinib + DC-CIK = 27, erlotinib = 27)	III and IV	Tumor lysate	Immature	All patients received erlotinib. The DC-CIK + erlotinib group in addition received 4 s.c. DC vaccinations and 5 i.v. CIK vaccinations within the erlotinib treatment. Patients received treatment cycles until disease progression or withdrawal from the study (≥ 2 cycles).	Circulating CD4 T cells, CD8 T cells and the CD4/CD8 ratio were significantly increased after erlotinib + DC-CIK treatment, while there were no differences in these parameters in the erlotinib only group.. PFS was significantly increased in the DC-CIK + erlotinib group compared to the erlotinib only group. There was no difference in OS between both treatment groups.
**Hu et al., Med. Oncol. (** 50 **)**	N = 27^a^	III and IV	Tumor lysate	Immature	Patients received pemetrexed chemotherapy followed by i.d. DC vaccination at day 12. Patients received multiple rounds of DC vaccination until disease progression (≥ 2 cycles) or up to a maximum of 6 rounds.	Primary endpoint was safety and combination therapy was shown safe. No clinical nor immunological effect could be determined, since no control group was available.
**Zhao et al., Exp. Ther. Med. (** 51 **)**	N = 157 (DC-CIK + chemotherapy = 79; chemotherapy = 78)	IIIA	–	Immature	All patients received surgery. Chemotherapy consisted of four cycles of gemcitabine and cisplatin. 2 i.v. DC-CIK vaccinations were administered after the second cycle and after the fourth cycle of chemotherapy in the DC-CIK + chemotherapy group.	The 3-year cumulative recurrence rate was significantly reduced in the DC-CIK + chemotherapy group. The 3-year cumulative survival was significantly increased in the DC-CIK + chemotherapy group.
**Zhu et al., Genet. Mol. Res. (** 52 **)**	N = 65 (DC-CIK + radio-/chemotherapy = 30; radio-/chemotherapy = 35)	IIIB	-	Unknown^b^	All patients received 4 cycles of docetaxel and cisplatin chemotherapy combined with a total dose of 60-70 Gy radiotherapy. The DC-CIK + radio/chemotherapy group received 4 rounds of 2 or 3 i.v. DC-CIK vaccinations in between the chemo- and radiotherapy cycles.	Patients in the DC-CIK + radio/chemotherapy group demonstrated significantly increased CD3 and CD4 T cells 4 weeks after treatment. This difference was not observed in the radio/chemotherapy group only. Patients in the DC-CIK + radio/chemotherapy group demonstrated significantly increased 6-months and 12-months OS compared to the radio-/chemotherapy alone group.
**Zhang et al., Oncol. Lett. (** 53 **)**	N = 507 (DC-CIK + standard therapy = 99; standard therapy = 408)	III and IV	NSCLC cell line lysate	Unknown^b^	Standard therapy consisted of surgery, chemotherapy, and radiotherapy. DCs were administered i.v. once a week for 3 weeks. In the first week of treatment, patients received i.v. CIK vaccinations once a day for 4 days. After 3 weeks, patients received i.d. DC vaccinations once a week for 3 weeks.	59 out of 97 patients from the combination therapy group demonstrated a DTH response (the control group was not tested). Patients who received DC-CIK showed significantly improved survival compared to patients who received standard therapy.
**Zhang et al., Radiot. Oncol. (** 54 **)**	N = 82 (DC-CIK + radiotherapy = 21; radiotherapy = 61)	III and IV	MUC-1 peptide	Unknown^b^	All patients received a total dose of 60-66 Gy radiotherapy. The DC-CIK + radiotherapy group received 4 s.c. DC vaccinations and 4 i.v. CIK vaccinations between radiotherapy fractions.	Peripheral blood of before and after treatment was available for 20 patients. No differences in circulating CD8 T cells, CD4 T cells and NK cells were observed between before and after treatment in both treatment groups. Patients in the DC-CIK + radiotherapy group showed a significantly increased PFS compared to the radiotherapy only group. No difference in OS between both treatment groups was observed.
**Zhao et al., Clin. Transl. Oncol. (** 55 **)**	N = 135 (DC-CIK = 45; chemotherapy = 40; DC-CIK + chemotherapy = 50	III and IV	–	Partly mature^c^	Chemotherapy consisted of pemetrexed or docetaxel. DC-CIK was administered i.v. daily for 3 days. Patients of all groups received ≥ 2 rounds of treatment.	In multivariate analysis, combination therapy of DC-CIK and chemotherapy was an independent prognostic factor for increased 1-year PFS and OS. There was no difference in 1-year OS between the DC-CIK only and the chemotherapy only group.

Studies are displayed in order of publication (old to new). DC-CIK therapy or DCs for DC vaccination were derived from autologous PBMCs. ^a^All patients failed gefitinib or erlotinib maintenance therapy. ^b^No established maturation method was used and no data that showed the maturation status of the DCs was available, ^c^showed a CD80^+^CD86^+^ population > 80% in their vaccine; SCC, squamous cell carcinoma; TNF-α, tumor necrosis factor α; CEA, carcinoembryonic antigen; i.v., intravenous; s.c., subcutaneous; MUC-1, Mucin-1; PFS, progression-free survival.

Importantly, all studies showed that combination therapy was safe and well-tolerated. From all studies that combined DC-based therapy with radio- and/or chemotherapy in NSCLC, four out of seven demonstrated improved OS in the combination therapy group compared to radiotherapy or chemotherapy alone. Two studies of combined DC-CIK therapy that showed no differences in OS between groups, did show improved disease-free survival (DFS) in the combination therapy group. Unfortunately, the study investigating the effect of chemotherapy and DC vaccination alone included no control group to compare treatment efficiency or clinical outcome.

In addition to chemotherapy and radiotherapy, one study investigated the combination of DC-CIK and the EGFR tyrosine kinase inhibitor erlotinib in patients with advanced stage NSCLC (**Table 2**) (49). This study demonstrated increased progression-free survival (PFS) in the combination therapy group, while OS did not differ between the groups. This synergistic effect is particularly interesting considering that EGFR-mutated NSCLC is insensitive to anti-PD-1/anti-PD-L1 therapy (15, 59, 60). Erlotinib is normally not combined with other systemic treatment, because it shows no benefit in survival to monotherapy, while toxicity potentially increases (61, 62).

Whereas the current DC-based monotherapy studies could not show clinical benefit in NSCLC patients, combinations with chemotherapy, radiotherapy, and targeted therapy showed to improve clinical outcome. However, in almost all combination studies CIK cells were administered simultaneously with the DC vaccine. Hence, whether the observed clinical advantage of combination therapy over the standard therapy is an effect of the DC vaccine, the CIK cells, or the combination of both cannot be discerned from those studies.

A combination of therapies that was not studied in NSCLC before is DC-based therapy and other immunotherapy, such as ICIs. Several studies have pointed out that when there is no anti-tumor immune response, ‘releasing the brakes’ by checkpoint inhibition will not lead to improved clinical results. Hence, a combination with an immune strategy that actively induces an anti-tumor immune response might improve therapy response rate (63). Vice versa, therapies that actively stimulate the immune response, often result in increased expression of immune checkpoint molecules and might therefore also benefit from a combination treatment with ICIs. In addition, in single-cell RNA sequencing data from NSCLC tissue a mature DC subset with high expression of regulatory molecules, such as PD-L1, was identified which could be targeted by anti-PD-L1 therapy (64).

The potential synergistic effect of ICIs and DC vaccination is currently examined in advanced stage melanoma patients. Accordingly, two studies showed that a combination strategy of anti-CTLA-4 and DC vaccination resulted in an improved clinical response compared to similar cohorts that received anti-CTLA-4 treatment alone, without causing additional toxicity (65–68). In addition, ICI therapy was shown to be effective in advanced stage melanoma patients with recurrent disease after adjuvant DC vaccination (69). Until date, no results are available of studies that examine whether the synergistic effect of combined ICI and DC-based therapy also applies for NSCLC.

## From Peripheral Immune Activation Towards a Local Response

An important question is whether the current administration route for DC vaccination in NSCLC can induce a tumor-specific immune response in the lungs. The most promising results from human DC-based therapies are achieved in melanoma. In these trials, DCs were injected in the skin and migrated to cutaneous lymph nodes in which maturated DCs can initiate an anti-tumor immune response. For melanoma, this is often in close proximity to the tumor. For NSCLC this same route of administration is chosen, although the tumor is located at a large distance from the cutaneous lymph nodes. This difference in environment of T cell activation might lead to a decreased amount of T cells that reach the tumor. This is illustrated in a pancreatic cancer mouse model in which intraperitoneal administration of a DC vaccine suppressed tumor growth and inhibited tumor progression to a larger extent compared to subcutaneous injections of the same DC vaccine (70). It might therefore be interesting to study the effect of DC-based therapy that is administered into the local lymph nodes. Although this is more invasive, it might induce a more locally effective anti-tumor immune response.

The local environment during T cell activation might not be the only element causing the suggested suboptimal lung T cell infiltration. In an inflammation mouse model, it was shown, that after local immunization with the immunogenic ovalbumin protein (OVA), DCs isolated from lung-draining mediastinal lymph nodes induced increased lung homing of CD4 T cells compared to DCs isolated from muscle-draining inguinal lymph nodes (71). The authors linked this increased ability to induce lung-homing of CD4 T cells to a CD24^+^ DC subset that is highly expressed in the mediastinal lymph node compared to the inguinal lymph node. To induce a local immune reaction in the lung, it therefore seems pivotal to specifically target this DC subset. Interestingly, in another inflammation mouse model it was demonstrated that this specific DC subset is probably not induced in the lung-draining lymph node itself, but rather in the lungs before lymph node migration (72). This is illustrated by an experiment in which DCs isolated from lung tissue, lung-draining lymph nodes, and other lymph nodes received antigen and were co-cultured with T cells *ex vivo*. DCs isolated from lung were superior at inducing lung-homing T cells, while there was no difference between DCs originating from lung-draining lymph nodes and other lymph nodes. This finding that lung-derived DCs induced superior homing of T cells to the lung was also confirmed in another mouse model of viral infection. In this model, mice were intranasally challenged with viral particles after which DCs were isolated from both lung and lung-draining lymph nodes at multiple time points after infection. Their results demonstrate that at 30 minutes after infection lung DCs were superior at inducing T cell homing compared to DCs originating from lung-draining lymph nodes, while at 24 hours after infection this difference was abolished. Since transport of soluble antigen from the lung towards the local lymph node already occurs within a few minutes after infection, this experiment validates that DCs that migrated from the lung are the main stimulants for lung-homing T cells. Although these are all pre-clinical data, a lung-derived DC subset paramount for optimal local tumor-specific T cell immune response might also be present in humans. DCs used for DC vaccination in NSCLC are not lung-derived. Therefore, means to equip DCs with an optimal capacity to induce lung-homing T cells should be developed.

## *In Vivo* Targeting of DCs

The clinical trials using DC-based therapy in NSCLC were performed with DCs that were earlier isolated from the patient’s peripheral blood, after which the DC vaccine was finalized in the lab. This procedure to construct DC vaccines has disadvantages: it is demanding for patients, laborious and expensive. For that reason, targeting DCs *in vivo* is a promising approach. Moreover, specific DC subsets in the tumor could be directly targeted when a monoclonal antibody directed against specific endocytic DC receptors would be used as guide for antigen delivery. For example, C-type lectin domain containing 9A (CLEC9A) on conventional type 1 DCs (cDC1s). Since CLEC9A is involved in cross-presentation, this specific targeting could also skew the antigen-processing towards this direction (73). This strategy of specific DC-targeting is not yet performed in humans, but some mouse studies show promising results.

In melanoma mouse models, receptors targeting among others, CLEC9A, CD11c, and DEC-205 (receptor on circulating DCs of mice and human, involved in cross-presentation) were bound to OVA (74–77). With concurrent activation, such as Polyinosinic:polycytidylic acid (poly I:C) and anti-CD40, the administered tumor antigen-receptor complex was shown to elicit effective immune responses and inhibit tumor growth. Hence, in the study using DEC-205 it was even demonstrated that *in vivo* vaccination showed larger inhibition of tumor growth compared to vaccination with *ex vivo* spleen-derived DCs which were also primed with OVA and maturated using anti-CD40 (74).

In the previous examples, antigens were chemically conjugated to a monoclonal antibody, or the DNA sequence of the antigen was genetically fused to the monoclonal antibody, while DC activation signals were injected separately. Currently, however, many studies focus on more complex manners of antigen delivery *in vivo* that could improve antigen uptake and DC activation efficiency. Examples are lipid vesicles or nanoparticles with surface-bound DC targeting receptors, and containing tumor antigens and DC activation signals (78). An important advantage of this technique is that the maturation signals are selectively delivered to the DCs. This is important because maturation molecules have been shown to have a tumor-supporting function when binding to other cells in the TME (79–81).

There are also studies that only focus on *in vivo* DC activation, without loading the DC with antigen. In a lymphoma mouse model, it is shown that intratumoral injection of TriMix mRNA, encoding costimulatory molecules CD70, CD40 ligand, and constitutively active toll-like receptor 4 (TLR4), induces systemic tumor-specific T cell responses independent of the co-delivery of tumor antigen (82). Moreover, in animal cancer models they show increased survival after injection of TriMix mRNA. The uptake of Trimix mRNA relies on the ability of DCs to rapidly and selectively internalize free RNA and avoids off-target effects. These results suggest that tumor-infiltrating DCs may have acquired antigen, and that the problem in their malfunctioning phenotype is rather the lack of sufficient activation signals in their surroundings.

## Conclusion

Nowadays, DC-based therapy in NSCLC is still at a developmental stage. The DC vaccination studies performed are all early-phase studies that demonstrated low toxicity of the treatment, but were underpowered to show clinical benefit. However, most of these clinical trials showed that DC vaccination can induce the desired immune response. The latter highlights the potential of DC-based therapy in NSCLC and encourages further research that could advance the peripheral immunological effect to a radiological response or improved survival. In particular, studies that examine whether the anti-tumor immune response in peripheral blood or skin could also be induced in the tumor environment could provide more insight. The immune-activating ability of DC vaccination and the low toxicity of treatment make this therapy an excellent candidate for combination with other anti-cancer treatment. Clinical success of combination therapies is illustrated by the results of combination studies of chemotherapy and/or radiotherapy and even targeted therapy with DC-CIK vaccination in NSCLC. Likewise, studies in melanoma demonstrated the synergizing effect of DC-based therapy and ICIs. Especially this latter combination with ICIs, which inhibit the immunosuppressive TME, could allow the optimal immune-activating potential of DC based therapy to be revealed in NSCLC.

## Author Contributions

IAEvdH wrote the manuscript. GFG and BP wrote and reviewed the manuscript. IJMdV and MMvdH reviewed the manuscript. All authors contributed to the article and approved the submitted version.

## Conflict of Interest

The authors declare that the research was conducted in the absence of any commercial or financial relationships that could be construed as a potential conflict of interest.

## References

[1] WHO Cancer. Geneva, Switzerland: WHO (2018). Available at: https://www.who.int/health-topics/cancer#tab=tab_1.

[2] de Sousa VML Carvalho L. Heterogeneity in Lung Cancer. Pathobiology (2018) 85(1–2):96–107. 10.1159/000487440 29635240

[3] Society, A. C Lung Cancer (2020). Available at: https://www.cancer.org/cancer/lung-cancer/about/key-statistics.html.

[4] Society, A. C Lung Cancer Survival Rates (2020). Available at: https://www.cancer.org/cancer/lung-cancer/detection-diagnosis-staging/survival-rates.html.

[5] NKR Cijfers - IKNL. (n.d). Available at: https://iknl.nl/nkr-cijfers?fs%7Cepidemiologie_id=506&fs%7Ctumor_id=1&fs%7Cregio_id=530&fs%7Cperiode_id=545%2C546%2C547%2C548%2C549%2C550%2C551%2C552%2C553%2C554%2C555%2C556%2C557%2C558%2C559%2C560%2C561%2C562%2C563%2C564%2C565%2C566%2C567%2C568%2C569%2C570%2C571%2C572%2C544%2C543%2C542%2C541&fs%7Cgeslacht_id=622&fs%7Cleeftijdsgroep_id=655&fs%7Cjaren_na_diagnose_id=665&fs%7Ceenheid_id=681&cs%7Ctype=line&cs%7CxAxis=periode_id&cs%7Cseries=epidemiologie_id&ts%7CrowDimensions=periode_id&ts%7CcolumnDimensions=&lang%7Clanguage=nl.

[6] Wang JB Huang X Li FR. Impaired Dendritic Cell Functions in Lung Cancer: A Review of Recent Advances and Future Perspectives. Cancer Commun (Lond) (2019) 39(1):43. 10.1186/s40880-019-0387-3 31307548 PMC6631514

[7] Brierley J Wittekind C Gospodarowicz MK. Tnm Classiﬁcation of Malignant Tumours. 8th edition. Chichester, UK: Union for International Cancer Control (UICC) (2016) p. 1–241.

[8] Larsen JE Cascone T Gerber DE Heymach JV Minna JD. Targeted Therapies for Lung Cancer: Clinical Experience and Novel Agents. Cancer J (2011) 17(6):512–27. 10.1097/PPO.0b013e31823e701a PMC3381956 22157296

[9] Asao T Takahashi F Takahashi K. Resistance to Molecularly Targeted Therapy in Non-Small-Cell Lung Cancer. Respir Invest (2019) 57(1):20–6. 10.1016/j.resinv.2018.09.001 30293943

[10] Garon EB Rizvi NA Hui R Leighl N Balmanoukian AS Eder JP. Pembrolizumab for the Treatment of Non–Small-Cell Lung Cancer. N Engl J Med (2015) 372(12):2018–28. 10.1056/nejmoa1501824 25891174

[11] Lawrence MS Stojanov P Polak P Kryukov GV Cibulskis K Sivachenko A. Mutational Heterogeneity in Cancer and the Search for New Cancer-Associated Genes. Nature (2013) 499(7457):214–8. 10.1038/nature12213 PMC3919509 23770567

[12] Riley JL. PD-1 Signaling in Primary T Cells. Immunol Rev (2009) 229(1):114–25. 10.1111/j.1600-065X.2009.00767.x PMC3424066 19426218

[13] Pesce S Greppi M Grossi F Del Zotto G Moretta L Sivori S. PD/1-PD-Ls Checkpoint: Insight on the Potential Role of NK Cells. Front Immunol (2019) 10:1242. 10.3389/fimmu.2019.01242 31214193 PMC6557993

[14] Francisco LM Salinas VH Brown KE Vanguri VK Freeman GJ Kuchroo VK. PD-L1 Regulates the Development, Maintenance, and Function of Induced Regulatory T Cells. J Exp Med (2009) 206(13):3015–29. 10.1084/jem.20090847 PMC2806460 20008522

[15] Reck M Rodriguez-Abreu D Robinson AG Hui R Csoszi T Fulop A. Pembrolizumab Versus Chemotherapy for PD-L1-Positive Non-Small-Cell Lung Cancer. N Engl J Med (2016) 375(19):1823–33. 10.1056/NEJMoa1606774 27718847

[16] FDA. FDA Approves Nivolumab Plus Ipilimumab for First-Line mNSCLC (PD-L1 Tumor Expression ≥1%). Silver Spring, USA: FDA (2020). Available at: https://www.fda.gov/drugs/resources-information-approved-drugs/fda-approves-nivolumab-plus-ipilimumab-first-line-mnsclc-pd-l1-tumor-expression-1.

[17] EMA. Annex I Summary of Product Characteristics (2020). Available at: https://www.ema.europa.eu/en/documents/product-information/opdivo-epar-product-information_en.pdf.

[18] Weinberg RA. The Biology of Cancer. 2nd ed. New York, USA: Ww Norton & co (2013).

[19] Nowicki TS Hu-Lieskovan S Ribas A. Mechanisms of Resistance to PD-1 and PD-L1 Blockade. Cancer J (United States) (2018) 24(1):47–53. 10.1097/PPO.0000000000000303 PMC5785093 29360728

[20] Steinman RM. The Dendritic Cell System and Its Role in Immunogenicity. Annu Rev Immunol (1991) 9:271–96. 10.1146/annurev.iy.09.040191.001415 1910679

[21] Condon TV Sawyer RT Fenton MJ Riches DW. Lung Dendritic Cells at the Innate-Adaptive Immune Interface. J Leukoc Biol (2011) 90(5):883–95. 10.1189/jlb.0311134 PMC3206474 21807741

[22] Cook PC MacDonald AS. Dendritic Cells in Lung Immunopathology. Semin Immunopathol (2016) 38(4):449–60. 10.1007/s00281-016-0571-3 PMC4896986 27256370

[23] Cheever MA Higano CS. Provenge (Sipuleucel-T) in Prostate Cancer: The First FDA-approved Therapeutic Cancer Vaccine. Clin Cancer Res (2011) 17(11):3520–6. 10.1158/1078-0432.CCR-10-3126 21471425

[24] Dillman RO Cornforth AN Depriest C McClay EF Amatruda TT de Leon C. Tumor Stem Cell Antigens as Consolidative Active Specific Immunotherapy: A Randomized Phase II Trial of Dendritic Cells Versus Tumor Cells in Patients With Metastatic Melanoma. J Immunother (2012) 35(8):641–9. 10.1097/CJI.0b013e31826f79c8 22996370

[25] Liau LM Ashkan K Tran DD Campian JL Trusheim JE Cobbs CS. First Results on Survival From a Large Phase 3 Clinical Trial of an Autologous Dendritic Cell Vaccine in Newly Diagnosed Glioblastoma. J Transl Med (2018) 16(1):142. 10.1186/s12967-018-1507-6 29843811 PMC5975654

[26] Ueda Y Itoh T Nukaya I Kawashima I Okugawa K Yano Y. Dendritic Cell-Based Immunotherapy of Cancer With Carcinoembryonic Antigen-Derived, HLA-A24-Restricted CTL Epitope: Clinical Outcomes of 18 Patients With Metastatic Gastrointestinal or Lung Adenocarcinomas. Int J Oncol (2004) 24(4):909–17. 10.3892/ijo.24.4.909 15010829

[27] Hirschowitz EA Foody T Kryscio R Dickson L Sturgill J Yannelli J. Autologous Dendritic Cell Vaccines for Non-Small-Cell Lung Cancer. J Clin Oncol (2004) 22(14):2808–15. 10.1200/JCO.2004.01.074 15254048

[28] Chang GC Lan HC Juang SH Wu YC Lee HC Hung YM. A Pilot Clinical Trial of Vaccination With Dendritic Cells Pulsed With Autologous Tumor Cells Derived From Malignant Pleural Effusion in Patients With Late-Stage Lung Carcinoma. Cancer (2005) 103(4):763–71. 10.1002/cncr.20843 15637694

[29] Hirschowitz EA Foody T Hidalgo GE Yannelli JR. Immunization of NSCLC Patients With Antigen-Pulsed Immature Autologous Dendritic Cells. Lung Cancer (2007) 57(3):365–72. 10.1016/j.lungcan.2007.04.002 PMC2063443 17509725

[30] Um SJ Choi YJ Shin HJ Son CH Park YS Roh MS. Phase I Study of Autologous Dendritic Cell Tumor Vaccine in Patients With Non-Small Cell Lung Cancer. Lung Cancer (2010) 70(2):188–94. 10.1016/j.lungcan.2010.02.006 20223553

[31] Perroud MW Jr. Honma HN Barbeiro AS Gilli SC Almeida MT Vassallo J. Mature Autologous Dendritic Cell Vaccines in Advanced Non-Small Cell Lung Cancer: A Phase I Pilot Study. J Exp Clin Cancer Res (2011) 30:65. 10.1186/1756-9966-30-65 21682877 PMC3135553

[32] Engell-Noerregaard L Kvistborg P Zocca M-B Pedersen AW Claesson MH Mellemgaard A. Clinical and Immunological Effects in Patients With Advanced Non-Small Cell Lung-Cancer After Vaccination With Dendritic Cells Exposed to an Allogeneic Tumor Cell Lysate. World J Vaccines (2013) 03(02):68–76. 10.4236/wjv.2013.32011

[33] Takahashi H Okamoto M Shimodaira S Tsujitani S Nagaya M Ishidao T. Impact of Dendritic Cell Vaccines Pulsed With Wilms’ Tumour-1 Peptide Antigen on the Survival of Patients With Advanced Non-Small Cell Lung Cancers. Eur J Cancer (2013) 49(4):852–9. 10.1016/j.ejca.2012.11.005 23245331

[34] Takahashi H Shimodaira S Ogasawara M Ota S Kobayashi M Abe H. Lung Adenocarcinoma may be a More Susceptive Subtype to a Dendritic Cell-Based Cancer Vaccine Than Other Subtypes of Non-Small Cell Lung Cancers: A Multicenter Retrospective Analysis. Cancer Immunol Immunother (2016) 65(9):1099–111. 10.1007/s00262-016-1872-z PMC11029687 27448677

[35] Ge C Li R Song H Geng T Yang J Tan Q. Phase I Clinical Trial of a Novel Autologous Modified-DC Vaccine in Patients With Resected NSCLC. BMC Cancer (2017) 17(1):884. 10.1186/s12885-017-3859-3 29268708 PMC5740508

[36] Li D He S. MAGE3 and Survivin Activated Dendritic Cell Immunotherapy for the Treatment of Non-Small Cell Lung Cancer. Oncol Lett (2018) 15(6):8777–83. 10.3892/ol.2018.8362 PMC5950540 29805617

[37] Emens LA Middleton G. The Interplay of Immunotherapy and Chemotherapy: Harnessing Potential Synergies. Cancer Immunol Res (2015) 3(5):436–43. 10.1158/2326-6066.CIR-15-0064 PMC5012642 25941355

[38] Reits EA Hodge JW Herberts CA Groothuis TA Chakraborty M Wansley EK. Radiation Modulates the Peptide Repertoire, Enhances MHC Class I Expression, and Induces Successful Antitumor Immunotherapy. J Exp Med (2006) 203(5):1259–71. 10.1084/jem.20052494 PMC3212727 16636135

[39] Di Blasio S Wortel IM van Bladel DA de Vries LE Duiveman-de Boer T Worah K. Human CD1c(+) DCs Are Critical Cellular Mediators of Immune Responses Induced by Immunogenic Cell Death. Oncoimmunology (2016) 5(8):e1192739. 10.1080/2162402X.2016.1192739 27622063 PMC5007971

[40] Strome SE Voss S Wilcox R Wakefield TL Tamada K Flies D. Strategies for Antigen Loading of Dendritic Cells to Enhance the Antitumor Immune Response. Cancer Res (2002) 62(6):1884–9. 11912169

[41] Goldszmid RS Idoyaga J Bravo AI Steinman R Mordoh J Wainstok R. Dendritic Cells Charged With Apoptotic Tumor Cells Induce Long-Lived Protective CD4 + and CD8 + T Cell Immunity Against B16 Melanoma. J Immunol (2003) 171(11):5940–7. 10.4049/jimmunol.171.11.5940 14634105

[42] Komorowski M Tisonczyk J Kolakowska A Drozdz R Kozbor D. Modulation of the Tumor Microenvironment by CXCR4 Antagonist-Armed Viral Oncotherapy Enhances the Antitumor Efficacy of Dendritic Cell Vaccines Against Neuroblastoma in Syngeneic Mice. Viruses (2018) 10(9):455. 10.3390/v10090455 PMC6165252 30149659

[43] Lamberti MJ Nigro A Mentucci FM Vittar NBR Casolaro V Col JD. Dendritic Cells and Immunogenic Cancer Cell Death: A Combination for Improving Antitumor Immunity. Pharmaceutics (2020) 12(3):256. 10.3390/pharmaceutics12030256 PMC7151083 32178288

[44] Eisendle K Weinlich G Ebner S Forstner M Reider D Zelle-Rieser C. Combining Chemotherapy and Autologous Peptide-Pulsed Dendritic Cells Provides Survival Benefit in Stage IV Melanoma Patients. JDDG - J German Soc Dermatol (2020) 18(11):1270–7. 10.1111/ddg.14334 PMC7756560 33197129

[45] Flieswasser T Van Loenhout J Freire Boullosa L Van den Eynde A De Waele J Van Audenaerde J. Clinically Relevant Chemotherapeutics Have the Ability to Induce Immunogenic Cell Death in Non-Small Cell Lung Cancer. Cells (2020) 9(6):1474. 10.3390/cells9061474 PMC7349161 32560232

[46] Zhong H Han B Tourkova IL Lokshin A Rosenbloom A Shurin MR. Low-Dose Paclitaxel Prior to Intratumoral Dendritic Cell Vaccine Modulates Intratumoral Cytokine Network and Lung Cancer Growth. Clin Cancer Res (2007) 13(18 Pt 1):5455–62. 10.1158/1078-0432.CCR-07-0517 PMC2474691 17875775

[47] Choi CW Jeong MH Park YS Son CH Lee HR Koh EK. Combination Treatment of Stereotactic Body Radiation Therapy and Immature Dendritic Cell Vaccination for Augmentation of Local and Systemic Effects. Cancer Res Treat (2019) 51(2):464–73. 10.4143/crt.2018.186 PMC6473298 29879758

[48] Zhong R Teng J Han B Zhong H. Dendritic Cells Combining With Cytokine-Induced Killer Cells Synergize Chemotherapy in Patients With Late-Stage Non-Small Cell Lung Cancer. Cancer Immunol Immunother (2011) 60(10):1497–502. 10.1007/s00262-011-1060-0 PMC11029021 21681372

[49] Shi SB Tang XY Tian J Chang CX Li P Qi JL. Efficacy of Erlotinib Plus Dendritic Cells and Cytokine-Induced Killer Cells in Maintenance Therapy of Advanced Non-Small Cell Lung Cancer. J Immunother (2014) 37(4):250–5. 10.1097/CJI.0000000000000015 24714359

[50] Hu RH Shi SB Qi JL Tian J Tang XY Liu GF. Pemetrexed Plus Dendritic Cells as Second-Line Treatment for Patients With Stage IIIB/IV Non-Small Cell Lung Cancer Who had Treatment With TKI. Med Oncol (2014) 31(8):63. 10.1007/s12032-014-0063-z 24958515

[51] Zhao M Li H Li L Zhang Y. Effects of a Gemcitabine Plus Platinum Regimen Combined With a Dendritic Cell-Cytokine Induced Killer Immunotherapy on Recurrence and Survival Rate of Non-Small Cell Lung Cancer Patients. Exp Ther Med (2014) 7(5):1403–7. 10.3892/etm.2014.1574 PMC3991503 24940447

[52] Zhu XP Xu YH Zhou J Pan XF. A Clinical Study Evaluating Dendritic and Cytokine-Induced Killer Cells Combined With Concurrent Radiochemotherapy for Stage IIIB Non-Small Cell Lung Cancer. Genet Mol Res (2015) 14(3):10228–35. 10.4238/2015.August.28.6 26345959

[53] Zhang L Yang X Sun Z Li J Zhu H Li J. Dendritic Cell Vaccine and Cytokine-Induced Killer Cell Therapy for the Treatment of Advanced Non-Small Cell Lung Cancer. Oncol Lett (2016) 11(4):2605–10. 10.3892/ol.2016.4273 PMC4812113 27073525

[54] Zhang L Xu Y Shen J He F Zhang D Chen Z. Feasibility Study of DCs/CIKs Combined With Thoracic Radiotherapy for Patients With Locally Advanced or Metastatic Non-Small-Cell Lung Cancer. Radiat Oncol (2016) 11:60. 10.1186/s13014-016-0635-5 27097970 PMC4839093

[55] Zhao Y Qiao G Wang X Song Y Zhou X Jiang N. Combination of DC/CIK Adoptive T Cell Immunotherapy With Chemotherapy in Advanced Non-Small-Cell Lung Cancer (NSCLC) Patients: A Prospective Patients’ Preference-Based Study (PPPS). Clin Transl Oncol (2019) 21(6):721–8. 10.1007/s12094-018-1968-3 30374838

[56] Wang S Wang X Zhou X Lyerly HK Morse MA Ren J. DC-CIK as a Widely Applicable Cancer Immunotherapy. Expert Opin Biol Ther (2020) 20(6):601–7. 10.1080/14712598.2020.1728250 32033522

[57] Schmeel LC Schmeel FC Coch C Schmidt-Wolf IG. Cytokine-Induced Killer (CIK) Cells in Cancer Immunotherapy: Report of the International Registry on CIK Cells (IRCC). J Cancer Res Clin Oncol (2015) 141(5):839–49. 10.1007/s00432-014-1864-3 PMC11824144 25381063

[58] Marten A Ziske C Schottker B Renoth S Weineck S Buttgereit P. Interactions Between Dendritic Cells and Cytokine-Induced Killer Cells Lead to an Activation of Both Populations. J Immunother (2001) 24(6):502–10. 10.1097/00002371-200111000-00007 11759073

[59] Borghaei H Paz-Ares L Horn L Spigel DR Steins M Ready NE. Nivolumab Versus Docetaxel in Advanced Nonsquamous Non–Small-Cell Lung Cancer. N Engl J Med (2015) 373(17):1627–39. 10.1056/nejmoa1507643 PMC5705936 26412456

[60] Rittmeyer A Barlesi F Waterkamp D Park K Ciardiello F von Pawel J. Atezolizumab Versus Docetaxel in Patients With Previously Treated Non-Small-Cell Lung Cancer (OAK): A Phase 3, Open-Label, Multicentre Randomised Controlled Trial. Lancet (2017) 389(10066):255–65. 10.1016/S0140-6736(16)32517-X PMC6886121 27979383

[61] Herbst RS Prager D Hermann R Fehrenbacher L Johnson BE Sandler A. Tribute: A Phase III Trial of Erlotinib Hydrochloride (OSI-774) Combined With Carboplatin and Paclitaxel Chemotherapy in Advanced Non-Small-Cell Lung Cancer. J Clin Oncol (2005) 23(25):5892–9. 10.1200/JCO.2005.02.840 16043829

[62] Gatzemeier U Pluzanska A Szczesna A Kaukel E Roubec J De Rosa F. Phase III Study of Erlotinib in Combination With Cisplatin and Gemcitabine in Advanced Non-Small-Cell Lung Cancer: The Tarceva Lung Cancer Investigation Trial. J Clin Oncol (2007) 25(12):1545–52. 10.1200/JCO.2005.05.1474 17442998

[63] Saxena M Bhardwaj N. Re-Emergence of Dendritic Cell Vaccines for Cancer Treatment. Trends Cancer (2018) 4(2):119–37. 10.1016/j.trecan.2017.12.007 PMC5823288 29458962

[64] Maier B Leader AM Chen ST Tung N Chang C LeBerichel J. A Conserved Dendritic-Cell Regulatory Program Limits Antitumour Immunity. Nature (2020) 580(7802):257–62. 10.1038/s41586-020-2134-y PMC7787191 32269339

[65] Ribas A Comin-Anduix B Chmielowski B Jalil J de la Rocha P McCannel TA. Dendritic Cell Vaccination Combined With CTLA4 Blockade in Patients With Metastatic Melanoma. Clin Cancer Res (2009) 15(19):6267–76. 10.1158/1078-0432.CCR-09-1254 PMC2765061 19789309

[66] Hodi FS O’Day SJ McDermott DF Weber RW Sosman JA Haanen JB. Improved Survival With Ipilimumab in Patients With Metastatic Melanoma. N Engl J Med (2010) 363(8):711–23. 10.1056/NEJMoa1003466 PMC3549297 20525992

[67] Wilgenhof S Corthals J Heirman C van Baren N Lucas S Kvistborg P. Phase II Study of Autologous Monocyte-Derived mRNA Electroporated Dendritic Cells (TriMixDC-MEL) Plus Ipilimumab in Patients With Pretreated Advanced Melanoma. J Clin Oncol (2016) 34(12):1330–8. 10.1200/JCO.2015.63.4121 26926680

[68] Eggermont AM Chiarion-Sileni V Grob JJ Dummer R Wolchok JD Schmidt H. Adjuvant Ipilimumab Versus Placebo After Complete Resection of High-Risk Stage III Melanoma (EORTC 18071): A Randomised, Double-Blind, Phase 3 Trial. Lancet Oncol (2015) 16(5):522–30. 10.1016/S1470-2045(15)70122-1 25840693

[69] van Willigen WW Bloemendal M Boers-Sonderen MJ de Groot JWB Koornstra RHT van der Veldt AAM. Response and Survival of Metastatic Melanoma Patients Treated With Immune Checkpoint Inhibition for Recurrent Disease on Adjuvant Dendritic Cell Vaccination. OncoImmunology (2020) 9(1):1738814. 10.1080/2162402X.2020.1738814 33457087 PMC7790511

[70] Yang J Eresen A Shangguan J Ma Q Zhang Z Yaghmai V. Effect of Route of Administration on the Efficacy of Dendritic Cell Vaccine in PDAC Mice. Am J Cancer Res (2020) 10(11):3911–9. PMC7716172 33294276

[71] Pejoski D Ballester M Auderset F Vono M Christensen D Andersen P. Site-Specific DC Surface Signatures Influence CD4+ T Cell Co-Stimulation and Lung-Homing. Front Immunol (2019) 10:1650. 10.3389/fimmu.2019.01650 31396211 PMC6668556

[72] Mikhak Z Strassner JP Luster AD. Lung Dendritic Cells Imprint T Cell Lung Homing and Promote Lung Immunity Through the Chemokine Receptor CCR4. J Exp Med (2013) 210(9):1855–69. 10.1084/jem.20130091 PMC3754856 23960189

[73] Schreibelt G Klinkenberg LJJ Cruz LJ Tacken PJ Tel J Kreutz M. The C-type Lectin Receptor CLEC9A Mediates Antigen Uptake and (Cross-)Presentation by Human Blood BDCA3+ Myeloid Dendritic Cells. Blood (2012) 119(10):2284–92. 10.1182/blood-2011-08-373944 22234694

[74] Bonifaz LC Bonnyay DP Charalambous A Darguste DI Fujii S Soares H. In Vivo Targeting of Antigens to Maturing Dendritic Cells Via the DEC-205 Receptor Improves T Cell Vaccination. J Exp Med (2004) 199(6):815–24. 10.1084/jem.20032220 PMC2212731 15024047

[75] Sancho D Mourao-Sa D Joffre OP Schulz O Rogers NC Pennington DJ. Tumor Therapy in Mice Via Antigen Targeting to a Novel, DC-Restricted C-Type Lectin. J Clin Invest (2008) 118(6):2098–110. 10.1172/JCI34584 PMC2391066 18497879

[76] Wei H Wang S Zhang D Hou S Qian W Li B. Targeted Delivery of Tumor Antigens to Activated Dendritic Cells Via CD11c Molecules Induces Potent Antitumor Immunity in Mice. Clin Cancer Res (2009) 15(14):4612–21. 10.1158/1078-0432.CCR-08-3321 19584156

[77] Kato M McDonald KJ Khan S Ross IL Vuckovic S Chen K. Expression of Human DEC-205 (CD205) Multilectin Receptor on Leukocytes. Int Immunol (2006) 18(6):857–69. 10.1093/intimm/dxl022 16581822

[78] Caminschi I Maraskovsky E Heath WR. Targeting Dendritic Cells In Vivo for Cancer Therapy. Front Immunol (2012) 3:13. 10.3389/fimmu.2012.00013 22566899 PMC3342351

[79] Huang B Zhao J Li H He KL Chen Y Chen SH. Toll-Like Receptors on Tumor Cells Facilitate Evasion of Immune Surveillance. Cancer Res (2005) 65(12):5009–14. 10.1158/0008-5472.CAN-05-0784 15958541

[80] Chiodoni C Iezzi M Guiducci C Sangaletti S Alessandrini I Ratti C. Triggering CD40 on Endothelial Cells Contributes to Tumor Growth. J Exp Med (2006) 203(11):2441–50. 10.1084/jem.20060844 PMC2118135 17043144

[81] Dölen Y Gileadi U Chen J-L Valente M Creemers JHA Van Dinther EAW. PLGA Nanoparticles Co-Encapsulating NY-ESO-1 Peptides and IMM60 Induce Robust CD8 and CD4 T Cell and B Cell Responses. Front Immunol (2021) 12:641703. 10.3389/fimmu.2021.641703 33717196 PMC7947615

[82] Van Lint S Renmans D Broos K Goethals L Maenhout S Benteyn D. Intratumoral Delivery of TriMix mRNA Results in T-Cell Activation by Cross-Presenting Dendritic Cells. Cancer Immunol Res (2016) 4(2):146–56. 10.1158/2326-6066.CIR-15-0163 26659303

